# The clinical course of hospitalized COVID-19 patients and aggravation risk prediction models: a retrospective, multi-center Korean cohort study

**DOI:** 10.3389/fmed.2023.1239789

**Published:** 2024-01-04

**Authors:** Min Kyong Moon, Hyeonjung Ham, Soo Min Song, Chanhee Lee, Taewan Goo, Bumjo Oh, Seungyeoun Lee, Shin-Woo Kim, Taesung Park

**Affiliations:** ^1^Department of Internal Medicine, Seoul National University College of Medicine, Seoul, Republic of Korea; ^2^Department of Internal Medicine, SMG-SNU Boramae Medical Center, Seoul, Republic of Korea; ^3^Interdisciplinary Program in Bioinformatics, Seoul National University, Seoul, Republic of Korea; ^4^Department of Family Medicine, Seoul National University College of Medicine, Seoul, Republic of Korea; ^5^Department of Family Medicine, SMG-SNU Boramae Medical Center, Seoul, Republic of Korea; ^6^Department of Mathematics & Statistics, Sejong University, Seoul, Republic of Korea; ^7^Department of Internal Medicine, School of Medicine, Kyungpook National University, Daegu, Republic of Korea; ^8^Department of Statistics, Seoul National University, Seoul, Republic of Korea

**Keywords:** COVID-19, disease progression, case fatality rate, statistical model, clinical decision support

## Abstract

**Background:**

Understanding the clinical course and pivotal time points of COVID-19 aggravation is critical for enhancing patient monitoring. This retrospective, multi-center cohort study aims to identify these significant time points and associate them with potential risk factors, leveraging data from a sizable cohort with mild-to-moderate symptoms upon admission.

**Methods:**

This study included data from 1,696 COVID-19 patients with mild-to-moderate clinical severity upon admission across multiple hospitals in Daegu-Kyungpook Province (Daegu dataset) between February 18 and early March 2020 and 321 COVID-19 patients at Seoul Boramae Hospital (Boramae dataset) collected from February to July 2020. The approach involved: (1) identifying the optimal time point for aggravation using survival analyses with maximally selected rank statistics; (2) investigating the relationship between comorbidities and time to aggravation; and (3) developing prediction models through machine learning techniques. The models were validated internally among patients from the Daegu dataset and externally among patients from the Boramae dataset.

**Results:**

The Daegu dataset showed a mean age of 51.0 ± 19.6 years, with 8 days for aggravation and day 5 being identified as the pivotal point for survival. Contrary to previous findings, specific comorbidities had no notable impact on aggravation patterns. Prediction models utilizing factors including age and chest X-ray infiltration demonstrated promising performance, with the top model achieving an AUC of 0.827 in external validation for 5 days aggravation prediction.

**Conclusion:**

Our study highlights the crucial significance of the initial 5 days period post-admission in managing COVID-19 patients. The identification of this pivotal time frame, combined with our robust predictive models, provides valuable insights for early intervention strategies. This research underscores the potential of proactive monitoring and timely interventions in enhancing patient outcomes, particularly for those at risk of rapid aggravation. Our findings offer a meaningful contribution to understanding the COVID-19 clinical course and supporting healthcare providers in optimizing patient care and resource allocation.

## Introduction

From the onset of the COVID-19 pandemic in the winter of 2019 until September 6, 2023, 770,437,327 confirmed cases occurred worldwide, including 6,956,900 deaths (1). Vaccines have facilitated a potential return to pre-pandemic conditions, yet significant challenges persist. These include the uneven global distribution of vaccine resources and the continual emergence of new SARS-CoV-2 variants, underscoring the ongoing nature of the battle against this virus.

The case fatality rates (CFRs) of COVID-19 vary, ranging from 0.3% to 9.4% across different countries (2, 3). Even in the same country, when an outbreak occurs during a certain period, it is higher in a specific group (4). For example, in New York City, the CFR in patients who were 65 years and older who did not receive mechanical ventilation was 26.6% (5). In the Lombardy region in Italy, the CFR in patients who were 64 years and older admitted to the intensive care unit was 36% (6). Such differences in mortality among countries may be explained by differences in patient characteristics, including age and comorbidities, and the availability of medical resources, such as beds, medical staff, ventilators, and medical oxygen therapy. Community treatment centers, introduced in the Republic of Korea in the early days of the COVID-19 pandemic, are considered a successful means to ensure that severely ill patients receive the necessary medical resources (7). Therefore, it is essential to understand the clinical severity of the disease in patients to prioritize care for those who either have elevated clinical severity or are at high risk of progressing to severe disease, thus ensuring that beds and medical resources are allocated appropriately.

In adapting to the changing COVID-19 landscape, it is critical to identify risk factors that contribute to the deteriorating prognosis of patients. Although several studies have reported risk factors such as older age, male sex, diabetes, obesity, hypertension, cardiovascular disease, chronic lung disease, cancer, and chronic kidney disease (CKD) (8–10), a consensus remains elusive. For example, a meta-analysis involving 45 studies and 18,300 patients revealed that while older age and diabetes were significantly associated with a higher risk of in-hospital mortality, other factors such as male sex, hypertension, and smoking did not demonstrate a significant impact (10). This highlights the need for further research to better understand the specific comorbidities that contribute to poor COVID-19 outcomes and the mechanisms by which these diseases influence the severity or fatality of the infection.

Furthermore, understanding COVID-19’s clinical trajectory remains central to enhancing patient monitoring. Numerous studies on the progression of COVID-19 have presented varied results regarding clinical events such as the onset of dyspnea and the duration of recovery (4). For instance, in the Republic of Korea, 98% of the patients who did not require supplemental oxygen on admission recovered by the 14th day (4). In contrast, the illness often intensified between days 4 and 10 in outpatient COVID-19 clinics in the United States, typically marked by the onset of dyspnea. Moreover, some patients experienced severe symptoms extending beyond the 14th day from the initial symptom onset (11). A separate study focusing on a cohort of 138 patients hospitalized in Wuhan due to COVID-19-induced pneumonia reported that a majority encountered dyspnea within the first 5 days after the onset of symptoms (12). This finding was echoed in another study wherein the median timeline to dyspnea was also identified as the 5th day, while the onset of acute respiratory distress syndrome (ARDS) was 8 days after symptom onset (13).

Recent advances in machine learning have brought significant insights to the understanding of COVID-19 severities. In a recent study, machine learning was employed to classify COVID-19 patients into distinct immune phenotype groups based on serum cytokine and antibody measurements, offering a novel approach to stratify patients at hospital admission for personalized therapy guidance (14). A convolutional neural network (CNN) model augmented with extreme gradient boosting (XGBoost) and a hybrid optimization algorithm was successfully employed to develop an automated image analysis framework for early COVID-19 detection in chest X-ray scans, achieving a remarkable classification accuracy of approximately 99.39% and demonstrating significant potential in addressing diagnostic challenges during the global pandemic (15). In a study conducted in northern Italy during the early months of the COVID-19 pandemic, a hybrid machine learning/deep learning model was developed to classify patients into non-ICU and ICU outcome categories based on clinical and CT image data, achieving a high probabilistic AUC of 0.949 and offering clinical decision support to medical professionals (16).

In this study, we conducted a detailed analysis of the clinical progression of hospitalized COVID-19 patients, drawing from a retrospective multi-center cohort study conducted across several hospitals in Korea. We hypothesized that identifying critical time points in the early stages of hospital admission for COVID-19 patients can significantly improve patient monitoring and outcomes. Therefore, the primary focus was to discover patterns in disease progression, with an emphasis on critical stages and outcomes. Prediction models based on machine learning were developed and evaluated to forecast patient aggravation, incorporating a range of clinical and demographic variables. Our findings are geared toward providing actionable insights that can aid in the optimization of treatment protocols and resource allocation in healthcare settings, ultimately contributing to more effective and personalized management of COVID-19 patients. The article is structured as follows: the Methods section provides a comprehensive account of the study’s COVID-19 patient data collection, detailing 1,696 patients in Daegu and 321 in Seoul. This section also delves into the statistical approaches used to identify the time point at which the survival rate difference between patients before and after this cut point is maximized. Moreover, the Methods section introduces the development of prediction models using machine learning techniques. The Results section outlines the demographic characteristics of the study cohort, the progression of the disease over time, its association with various comorbidities, and assesses the prediction models. Lastly, the Discussion and Conclusions sections offer an interpretation of these findings, emphasizing the critical 5 days period following hospital admission and its significance for clinical intervention strategies.

## Methods

### Study design and data collection

A retrospective cohort study of patients with COVID-19 was conducted. The primary dataset, referred to as the “Daegu dataset,” consisted of multi-center clinical records registered by the Korea Disease Control and Prevention Agency. These records include clinical and demographic data of COVID-19 patients hospitalized across 10 different hospitals in Daegu-Kyungpook Province, Republic of Korea, from February 18 to early March 2020.

Of the 2,254 patients whose clinical data were available, 1,970 patients remained after excluding those without recorded discharge or death dates. The sample was further refined to 1,696 patients demonstrating mild-to-moderate clinical severity upon admission (Supplementary Figure S1). The clinical status of each patient was monitored daily. Details of the clinical severity criteria are described in the “Outcome definition” section.

In addition to the Daegu dataset, an external validation dataset was collected and curated, referred to as the “Boramae dataset.” This dataset contains the records of 321 patients from the Seoul Boramae Hospital, collected from February to July 2020, and closely aligns with the period of the Daegu dataset. A consistent set of variables was maintained across both datasets, except for the diastolic blood pressure (DBP) and autoimmune disease variable, ensuring high compatibility and facilitating comparative analysis.

### Demographics and clinical characteristics

A total of 34 and 32 clinical variables were recorded in the Daegu and Boramae datasets, respectively, including demographics (age and sex), initial vital signs (body temperature, blood pressure, heart rate, and respiratory rate), body mass index (BMI), smoking history, chest X-ray infiltration, symptoms (cough, sputum, sore throat, rhinorrhea, myalgia, fatigue or malaise, shortness of breath, headache, altered consciousness or confusion, vomiting, nausea, and diarrhea), and comorbidities [diabetes mellitus, heart failure, hypertension, asthma, malignancy, autoimmune disease, dementia, chronic liver disease (CLD), cardiovascular disease, CKD, chronic neurological disease (CND), chronic obstructive pulmonary disease (COPD), and chronic hematological disease (CHD)]. These variables were recorded upon admission. Any clinical variables with missing values were excluded. After exclusion, 27 of these variables were binary, presented as frequency per group, and 7 were continuous, summarized in terms of the mean and standard deviation per group. Tables 1, 2 illustrate the detailed demographics and clinical characteristics of the patients from the Daegu and Boramae datasets, respectively.

**Table 1:** Baseline demographics and clinical characteristics of the Daegu dataset.

Characteristics	Total (*N* = 1,696)	Non-aggravated (*N* = 1,506)	Aggravated (*N* = 190)	*p*-value
Age (years)	51 ± 19.6	48.7 ± 18.8	69.6 ± 15.1	<0.001
Male, *n* (%)	550/1696 (32.4)	478/1506 (31.7)	72/190 (37.9)	0.1
Body mass index (kg/m^2^)	23.4 ± 3.8	23.3 ± 3.9	23.9 ± 3.6	0.078
Current smoker, *n* (%)	93/1346 (6.9)	81/1189 (6.8)	12/157 (7.6)	0.737
Chest X-ray infiltration, n (%)	630/1661 (37.9)	509/1471 (34.6)	121/190 (63.7)	<0.001
*Vital signs on admission*				
Body temperature (°C)	37.1 ± 0.6	37.1 ± 0.5	37.4 ± 0.8	<0.001
Systolic blood pressure (mmHg)	135.3 ± 20	134.9 ± 19.6	137.8 ± 22.4	0.09
Diastolic blood pressure (mmHg)	81.9 ± 12.5	82 ± 12.5	81.3 ± 13	0.541
Heart rate (beats/min)	87.8 ± 15.1	88 ± 15.1	86.1 ± 14.7	0.083
Respiration rate (breaths/min)	20 ± 3.7	20 ± 3.9	20.3 ± 1.9	0.003
*Symptoms on admission*				
Cough	652/1358 (48)	563/1170 (48.1)	89/188 (47.3)	0.875
Sputum	507/1357 (37.4)	441/1170 (37.7)	66/187 (35.3)	0.569
Sore throat	197/1339 (14.7)	175/1155 (15.2)	22/184 (12)	0.313
Rhinorrhea	155/1328 (11.7)	137/1144 (12)	18/184 (9.8)	0.458
Myalgia	316/1306 (24.2)	271/1128 (24)	45/178 (25.3)	0.707
Fatigue/malaise	42/1317 (3.2)	32/1141 (2.8)	10/176 (5.7)	0.061
Shortness of breath	187/1337 (14)	148/1156 (12.8)	39/181 (21.5)	0.003
Headache	325/1340 (24.3)	293/1158 (25.3)	32/182 (17.6)	0.025
Altered consciousness/confusion	2/1309 (0.2)	0/1133 (0)	2/176 (1.1)	0.018
Vomiting/nausea	86/1322 (6.5)	75/1141 (6.6)	11/181 (6.1)	1
Diarrhea	201/1325 (15.2)	179/1145 (15.6)	22/180 (12.2)	0.264
*Comorbidities*				
Diabetes mellitus	228/1680 (13.6)	167/1497 (11.2)	61/183 (33.3)	<0.001
Heart failure	22/1625 (1.4)	9/1456 (0.6)	13/169 (7.7)	<0.001
Hypertension	395/1685 (23.4)	304/1500 (20.3)	91/185 (49.2)	<0.001
Asthma	43/1603 (2.7)	39/1440 (2.7)	4/163 (2.5)	1
Malignancy	60/1595 (3.8)	44/1427 (3.1)	16/168 (9.5)	<0.001
Autoimmune disease	10/1256 (0.8)	8/1092 (0.7)	2/164 (1.2)	0.628
Dementia	55/1253 (4.4)	22/1088 (2)	33/165 (20)	<0.001
Chronic liver disease	25/1608 (1.6)	19/1434 (1.3)	6/174 (3.4)	0.045
Cardiovascular disease	71/1636 (4.3)	47/1464 (3.2)	24/172 (14)	<0.001
Chronic kidney disease	20/1597 (1.3)	10/1431 (0.7)	10/166 (6)	<0.001
Chronic neurological disease	5/1581 (0.3)	1/1419 (0.1)	4/162 (2.5)	<0.001
Chronic obstructive pulmonary disease	20/1614 (1.2)	15/1445 (1)	5/169 (3)	0.05
Chronic hematological disease	10/1253 (0.8)	7/1092 (0.6)	3/161 (1.9)	0.127

^*^Data are presented as mean ± standard deviation for continuous variables or frequency (proportion) for dichotomous variables.

**Table 2:** Baseline demographics and clinical characteristics of the Boramae dataset.

Characteristics	Total (*N* = 321)	Non-Aggravated (*N* = 278)	Aggravated (*N* = 43)	*p*-value
Age (years)	47 ± 18	45 ± 17	64 ± 14	<0.001
Male, *n* (%)	155/321 (48)	133/278 (48)	22/43 (51)	0.7
Body mass index (kg/m^2^)	24.3 ± 3.8	24.0 ± 3.8	25.8 ± 3.4	<0.001
Current smoker, *n* (%)	47/321 (15)	45/278 (6.8)	2/43 (4.7)	0.046
Chest X-ray infiltration, *n* (%)	134/321 (42)	104/278 (37)	30/43 (70)	<0.001
*Vital signs on admission*				
Body temperature (°C)	37.21 ± 0.86	37.22 ± 0.86	37.15 ± 0.85	0.5
Systolic blood pressure (mmHg)	136 ± 18	135 ± 18	142 ± 21	0.10
Heart rate (beats/min)	88 ± 13	88 ± 13	91 ± 14	0.2
Respiration rate (breaths/min)	18 ± 1	18 ± 1	19 ± 1	0.6
*Symptoms on admission*				
Cough	111/321 (35)	94/278 (34)	17/43 (40)	0.5
Sputum	58/321 (18)	51/278 (18)	7/43 (16)	0.7
Sore throat	62/321 (19)	56/278 (20)	6/43 (14)	0.3
Rhinorrhea	17/321 (5.3)	17/278 (6.1)	0/43 (0)	0.14
Myalgia	43/321 (13)	36/278 (13)	7/43 (16)	0.6
Fatigue/malaise	9/321 (2.8)	3/278 (1.1)	6/43 (14)	<0.001
Shortness of breath	4/321 (1.2)	3/278 (1.1)	1/43 (2.3)	0.4
Headache	30/321 (9.3)	28/278 (10)	2/43 (4.7)	0.4
Altered consciousness/confusion	0/321 (0)	0/278 (0)	0/43 (0)	
Vomiting/nausea	5/321 (1.6)	4/278 (1.4)	1/43 (2.3)	0.5
Diarrhea	9/321 (2.8)	7/278 (2.5)	2/43 (4.7)	0.3
*Comorbidities*				
Diabetes mellitus	27/321 (8.4)	15/278 (5.4)	12/43 (28)	<0.001
Heart failure	6/321 (1.9)	3/278 (1.1)	3/43 (7.0)	0.034
Hypertension	63/321 (20)	39/278 (14)	24/43 (56)	<0.001
Asthma	2/321 (0.6)	2/278 (0.7)	0/43 (0)	>0.9
Malignancy	7/321 (2.2)	7/278 (2.5)	0/43 (0)	0.6
Dementia	12/321 (3.7)	5/278 (1.8)	7/43 (16)	<0.001
Chronic liver disease	2/321 (0.6)	2/278 (0.7)	0/43 (0)	>0.9
Cardiovascular disease	2/321 (0.6)	1/278 (0.4)	1/43 (2.3)	
Chronic kidney disease	1/321 (0.3)	1/278 (0.4)	0/43 (0)	>0.9
Chronic neurological disease	1/321 (0.3)	1/278 (0.4)	0/43 (0)	>0.9
Chronic obstructive pulmonary disease	0/321 (0)	0/278 (0)	0/43 (0)	
Chronic hematological disease	1/321(0.3)	0/278(0)	1/43 (2.3)	0.13

### Outcome definition

In this study, the primary outcome was time to aggravation of mild-to-moderate clinical severity following admission. Aggravation was defined as the time point when patients with mild-to-moderate severity progressed to severe or more, according to the following definitions of clinical severity. The time to aggravation was calculated from the date of admission to that of aggravation. The clinical severity of hospitalized patients was defined as follows: (1) mild: body temperature below 37.5°C, presence of any symptoms, but no demonstration of pneumonia; (2) moderate: demonstration of fever (body temperature ≥37.5°C) or pneumonia (diagnosis by a clinician), but no need for oxygenation therapy; (3) severe: pneumonia diagnosis by a clinician and need for additional oxygenation therapies (nasal prong, facial mask, or high-flow oxygenation therapy), and (4) critical: pneumonia diagnosis by a clinician and need for mechanical ventilation therapies, extracorporeal membrane oxygenation, or death (17). In the initial phase of the study (from February 18, 2020, to early March 2020), all patients, including asymptomatic ones, were admitted to hospitals in Daegu-Kyungpook Province (18), indicating the possible inclusion of asymptomatic patients with mild severity. In this study, “death” refers to fatalities that occurred among confirmed COVID-19 cases, unless there is a clearly identified alternative cause of death that is not related to COVID-19. CFR is defined as the proportion of deaths within the utilized dataset.

Prediction models were built focusing on two outcomes: whether a patient aggravated within 5 days post-admission (termed “5 days aggravation”) and whether a patient aggravated at any point during their hospital stay (termed “eventual aggravation”). The same definitions for these outcomes were applied in analyzing the Boramae dataset, which also contains information on patients’ conditions both within the first 5 days and throughout their entire hospital stay.

### Statistical analysis

#### Demographics

In analyzing differences in demographics and clinical characteristics between aggravated and non-aggravated patients, Fisher’s exact test was utilized for binary variables where categories had expected frequencies below 5. For categories with expected frequencies above 5, Pearson’s chi-square test was applied. Continuous variables were analyzed using the Wilcoxon rank-sum test, given that many of them exhibited non-normal distributions.

#### Survival analyses to determine an aggravation cut point

To determine the cut point for the aggravation time, which gives the largest survival rate difference between two groups (aggravated before the cut point days vs. aggravated after the cut point days), survival analyses with different cut points were conducted. The time to aggravation per patient was calculated based on the patient’s daily clinical status. Survival time was assessed from the date of hospital admission until either the date of death or the end of hospitalization. Patients who did not succumb during the hospitalization period were treated as censored cases. To determine a cut point for the aggravation that maximally separates the survival curves, a maximally selected rank statistics analysis was conducted (19, 20). This technique uses the log-rank statistic of observed data to find the most significant split in continuous variables, here implemented to identify the optimal time-to-aggravation cut point wherein the survival curves diverge maximally. The log-rank statistic guided the discernment of this aggravation cut point by maximizing the difference between the survival curves. This method of analysis was facilitated using the maxstat R package.

#### Association between comorbidities and time to aggravation

To assess the association between the time to aggravation and pre-existing comorbidities, additional survival analyses with different comorbidities were conducted, and the corresponding aggravation probabilities were estimated. The time to aggravation was defined as ranging from the date of hospital admission to the occurrence of aggravation. Only 190 patients who were aggravated during hospitalization were selected. Patients with comorbidities were investigated using surveys; individuals who did not participate were omitted from the analysis due to the lack of information. To ensure a robust analysis, only comorbidities with a sufficient number of aggravated patients (>30 individuals) were investigated separately. These included diabetes (61 individuals), hypertension (91 individuals), and dementia (33 individuals). Additionally, two comparative groups were established: a group with any one of the identified comorbidities and a control group consisting of 47 patients who reported no comorbidities.

To investigate the effect of underlying disease on the time to aggravation, the aggravation rates were calculated and visualized with a Kaplan–Meier curve for the two groups, with and without the underlying disease. Furthermore, the hazard ratio of aggravation was estimated using a Cox proportional hazards model, incorporating adjustments for potential confounding factors such as age and sex. This approach allowed inferring the relative risk of aggravation for individuals with the specified comorbidities compared to those without.

### Development and evaluation of the prediction model

Prediction models were developed to distinguish between aggravated and non-aggravated patients using various approaches, including logistic regression (LR), random forest (RF), support vector machine (SVM), k-nearest neighbor (KNN), extreme gradient boosting (XGBoost), and deep neural network (DNN). Model performance was evaluated using the area under the curve (AUC) of the receiver operating characteristic (ROC) curve. For RF, SVM, KNN, XGBoost, and DNN, hyperparameters were selected using a grid search. Each model was evaluated using fivefold cross-validation (CV), and the mean value of the performance measures was reported for the Daegu dataset (internal validation). For external validation using the Boramae dataset, parameters and hyperparameters selected from the Daegu dataset were used, and performance measure was reported using the total dataset. The ratio of aggravated patients to non-aggravated patients was stratified to have the same ratio per fold. Continuous predictors were standardized to maintain the same scale. Predictive marker sets were selected using the least absolute shrinkage and selection operator (LASSO) and stepwise regression methods. For LASSO regression, the tuning parameter value was selected using a fivefold CV. For stepwise regression, predictive markers were selected to maximize the validation performance measure (i.e., AUC). Patients with missing values were excluded from the predictive marker selection.

We began by using maximally selected statistics to establish a critical threshold for identifying patient aggravation. Following this, the study analyzed the associations of comorbidities with patient aggravation patterns, offering insights into the progression of COVID-19 under different health conditions. Predictive models were then developed to determine the likelihood of aggravation in patients, addressing both a 5 days period and the broader course of the disease.

The predictive marker selection using LASSO and stepwise regression was performed using R software (version 4.0.5). Prediction models, except for DNN, were built using R software (version 4.0.5). Prediction models for the DNN were built using Keras (version 2.4.3) with the TensorFlow backend (version 2.4.1).

## Results

### Demographics and clinical characteristics

We analyzed a total of 1,696 patients with mild-to-moderate clinical severity upon hospital admission due to COVID-19 from the Daegu dataset. This primary dataset was supplemented with the Boramae dataset, comprising 321 patients. Detailed information regarding the demographic and clinical characteristics of the individuals involved in the study is displayed in Tables 1, 2.

The Daegu dataset had a mean age of 51.0 ± 19.6 years, with 32.4% (550 patients) being male. The Boramae dataset recorded a mean age of 47 ± 18 years and comprised 155 (48%) male patients. To investigate the potential heterogeneity in clinical data across different hospitals, principal component analysis (PCA) was conducted, as shown in Supplementary Figure S2. This figure includes a PCA plot that incorporates all variables from participating hospitals for which complete datasets were available (Supplementary Figure S2A) and another that differentiates between the two major hospitals from the Daegu dataset, the Boramae dataset, and the rest (Supplementary Figure S2B). The PCA plots show that even though data were collected from multiple hospitals, no distinct clustering patterns emerged across the different institutions.

### The clinical course of aggravated patients

**Figure 1:**
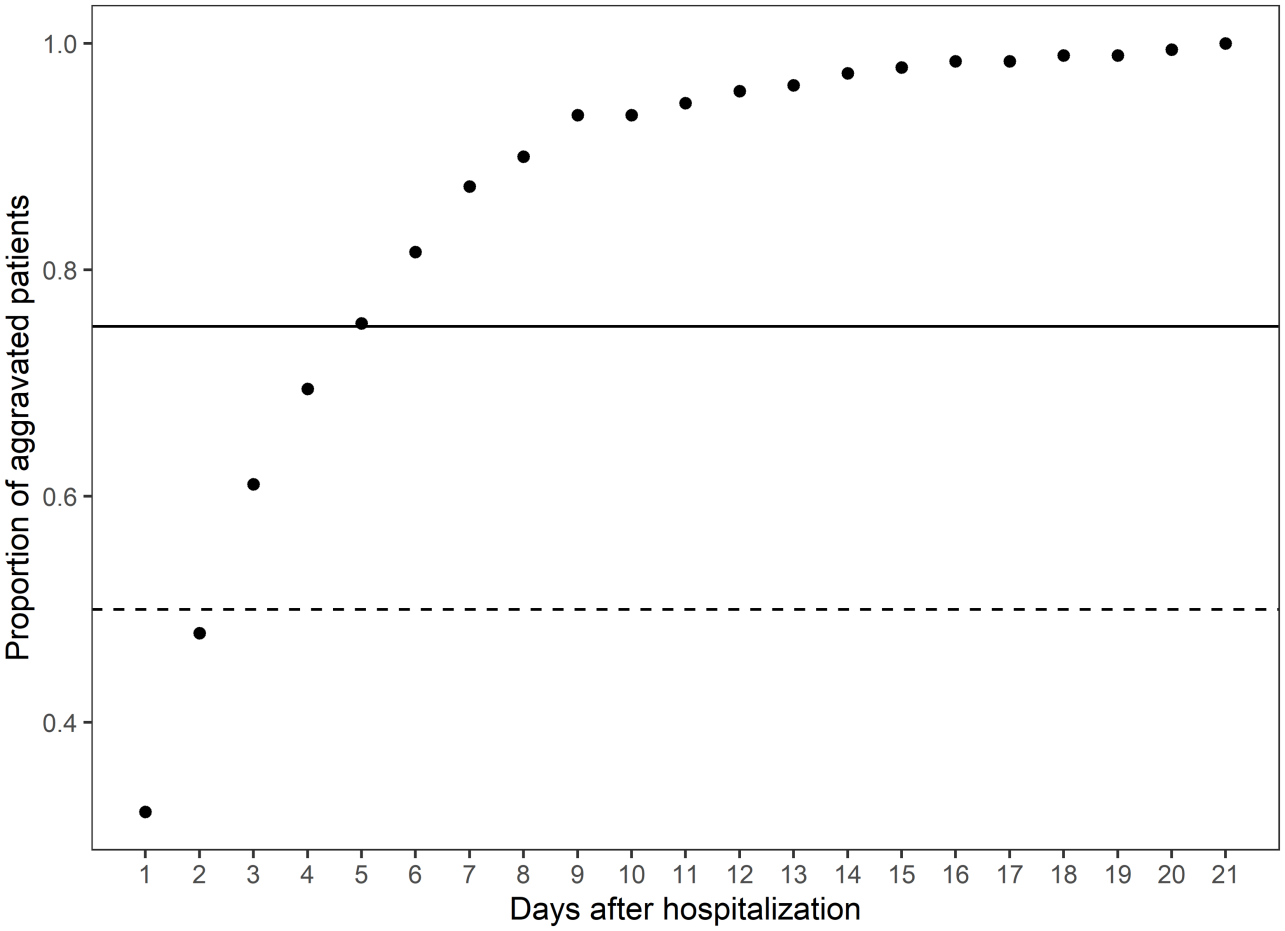
The cumulative proportion of aggravated patients on the days after hospitalization. The *x*-axis indicates the days after hospitalization. The *y*-axis indicates the cumulative proportion of aggravated patients at the specific time over all aggravated patients. The solid horizontal line indicates the 75th percentile of the aggravated patients, and the dashed line indicates the 50th percentile of the aggravated patients. The total number of aggravated patients is 190.

**Figure 2:**
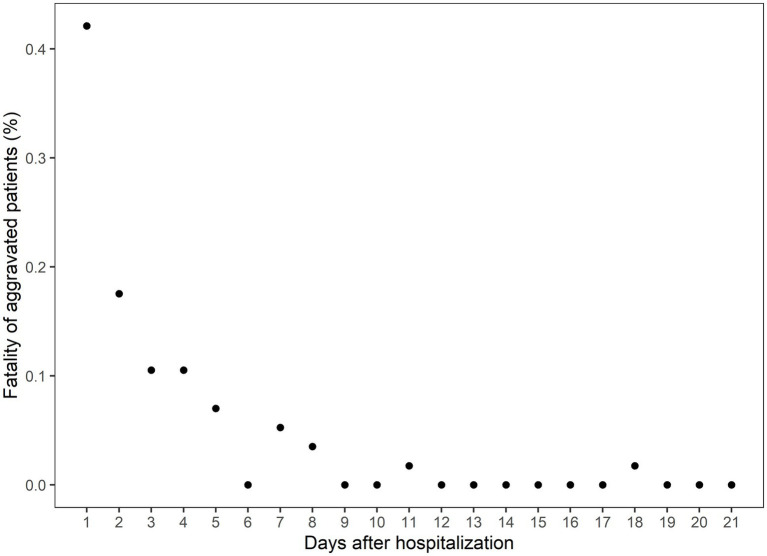
The case fatality rate among the aggravated patients on the days after hospitalization. The *x*-axis indicates the days after hospitalization, and the *y*-axis indicates the percentage of eventual deaths among the aggravated patients.

The CFR of the Daegu dataset was 3.4%. Out of all patients exhibiting mild-to-moderate clinical severity, 11.2% (190 individuals) experienced aggravation, with 30% of this subset (57 individuals) subsequently succumbing to the illness. A closer examination of the data revealed the following trend: nearly half of the aggravation, 47.89%, emerged within the first 2 days of hospitalization; this proportion increased to 75.26% by day 5 and reached 90% by day 8 (Figure 1). Fatality rates corresponded with earlier aggravation times (Figure 2). A majority of the deaths were concentrated among patients who aggravated within a span of 5 to 8 days post-hospitalization. Additionally, 86.2% of total fatalities (57 individuals) occurred among patients who aggravated within 5 days of hospitalization. Similarly, 94.8% of the overall fatality rate was observed among patients who aggravated within the first 8 days of hospitalization.

### Identification of the aggravation cut point

**Figure 3:**
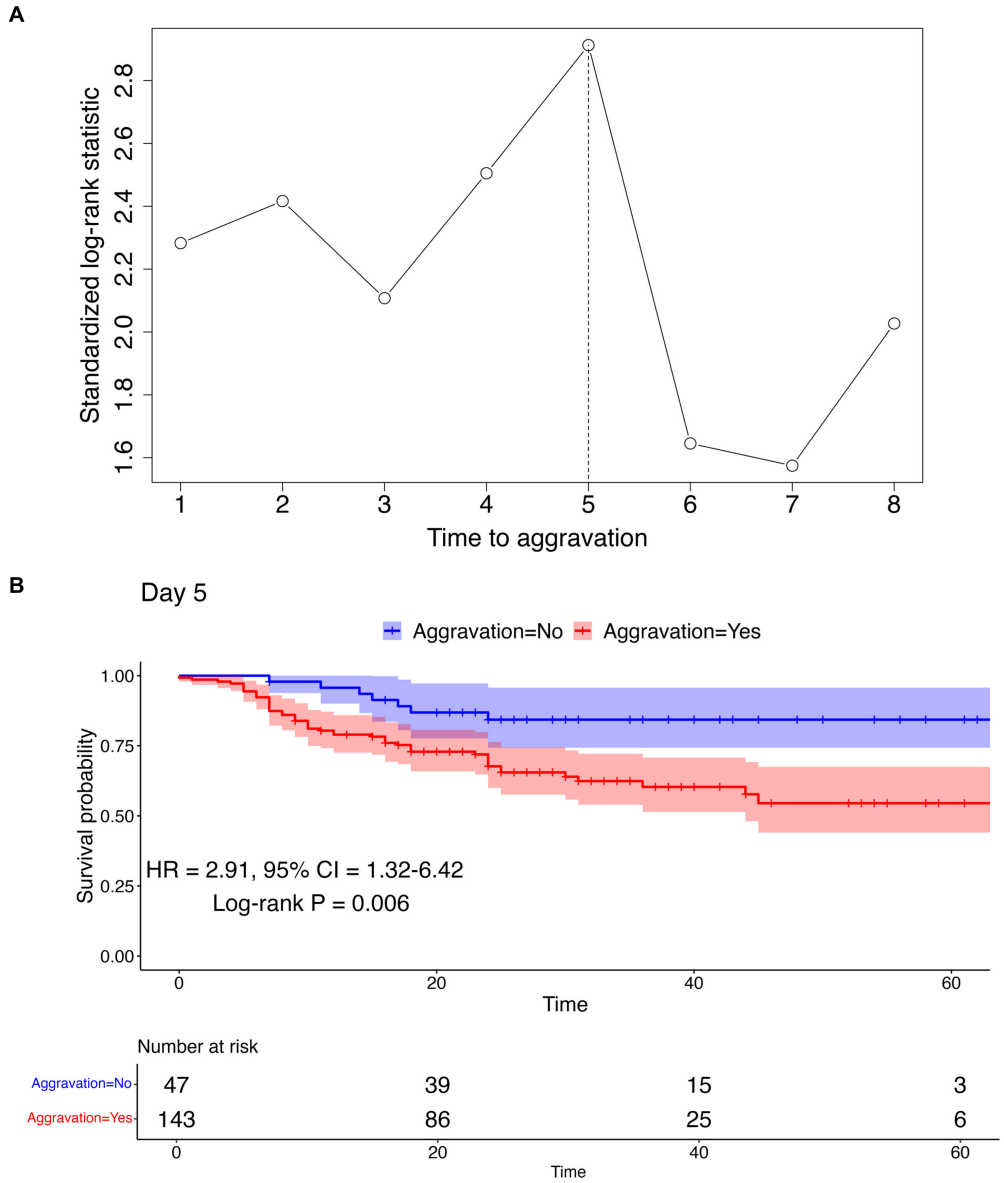
Cut point determination and confirmation using maximally selected rank statistics analysis and survival analysis. **(A)** Maximally selected rank statistics analysis result. The vertical dotted line represents the optimal cut point maximizing the standardized log-rank statistic. **(B)** Kaplan–Meier curve using the cut point of day 5 to differentiate aggravation and non-aggravation. The log-rank test *p*-value, HR, and 95% CI are represented below the curve. Kaplan–Meier curves for other cut points can be found in Supplementary Figure S3.

To determine the optimal cut point of aggravation, at which the survival rates between aggravated and non-aggravated patients are maximally differentiated, survival analyses were conducted employing a range of aggravation cut points. The time to death ranged from 0 to 67 days with the median at 24 days. Utilizing a maximally selected rank statistics analysis, a plot of cut points for the time to aggravation was constructed, as shown in Figure 3A; Supplementary Figure S3. The peak observed on day 5 in the standardized log-rank statistic pinpointed a maximal differentiation in survival rates when comparing patients who aggravated before and after the 5 days marker. To verify the difference in survival between the two groups, Kaplan–Meier curves were arrayed, and Cox PH regression and log-rank tests were conducted. The log-rank test revealed a statistically significant difference between the groups, with a *p*-value of 0.006. Furthermore, the Cox PH regression yielded a hazard ratio (HR) of 2.91, with a 95% confidence interval ranging from 1.32 to 6.42. The nearly non-overlapping confidence intervals between the two groups further support this significance, as illustrated in Figure 3B.

### Aggravation pattern according to comorbidities

**Figure 4:**
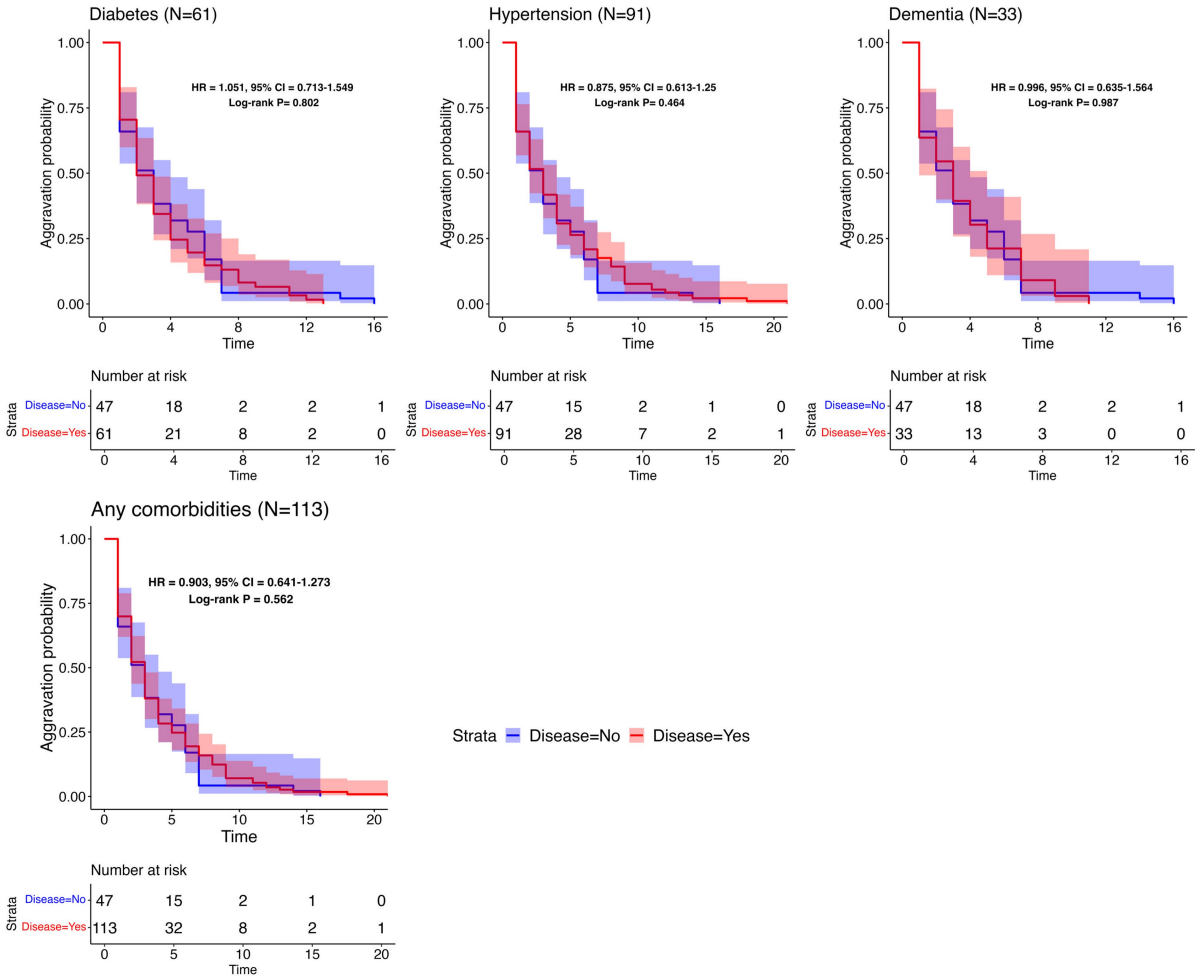
Survival analysis results per common comorbidity. Kaplan–Meier plots for the time to aggravation in the specific underlying disease group and the control group. The number of patients with each disease is represented in parentheses. The same control group without any comorbidities (*N* = 47) was used throughout the comparisons. The aggravation time for the control group ranges from 1 to 16 days, with a median of 3 days.

To determine the relationship between the time to aggravation and pre-existing comorbidities, additional analyses were undertaken using Kaplan–Meier plots. These plots were used to visualize potential differences between two groups: aggravated patients with a specific underlying disease and controls without any comorbidities. As visualized in Figure 4, each plot features log-rank test *p*-values, HR, and CI. The analyses did not reveal a significant distinction in aggravation patterns between individuals with a specified underlying disease and those without it. The last plot in Figure 4 illustrates a comparison between patients with at least one reported comorbidity and those without any disease, depicting the Kaplan–Meier curves for both groups. Since every patient in this analysis experienced aggravation, there were no censored cases, leading all Kaplan–Meier plots to ultimately approach zero. Supplementary Figure S4 contains the survival analysis results for less common comorbidities (with *N* ≥ 10).

### Prediction models for classifying 5 days aggravation and eventual aggravation

In this analysis, the Daegu dataset was utilized for internal validation and included 678 patients. For external validation, the Boramae dataset, comprising 321 patients, was employed. We utilized variable selection methods such as LASSO with 1 standard error (1se), LASSO with minimum criteria (min), and stepwise selection to identify predictive markers for two types of aggravation: 5 days aggravation and eventual aggravation. Key variables consistently identified by both methods included age, chest X-ray infiltration, heart failure, and chronic kidney disease (CKD). The selected variables and the performance of the models are detailed in Tables 3, 4. Supplementary Figure S5 offers an additional visual representation of model performance.

**Table 3:** Prediction model performance and selected predictive markers for 5 days aggravation.

Predictive marker selection method		Number of predictors	Selected predictive markers	Model	Internal validation AUC	External validation AUC
LASSO^*^	Min	11	Age + Body temperature + Chest X-ray infiltration + Diabetes + Heart failure + CKD	LR	0.786	0.796
				RF	0.786	0.772
				SVM	0.787	0.785
				KNN	0.677	0.650
				XGBoost	0.727	0.749
				DNN	0.802	0.732
	1se	4	Age + Chest X-ray infiltration + Heart failure + CKD	LR	0.789	0.827
				RF	0.713	0.737
				SVM	0.783	0.822
				KNN	0.710	0.678
				XGBoost	0.730	**0.829**
				DNN	0.793	0.822
Stepwise regression		10	Age + Chest X-ray infiltration + Body temperature + Smoking + CKD + CND + Malignancy + Heart failure + Myalgia + CLD	LR	**0.828**	0.785
				RF	0.793	0.743
				SVM	0.823	0.784
				KNN	0.706	0.647
				XGBoost	0.789	0.733
				DNN	0.820	0.747

^*^LASSO, least absolute shrinkage and selection operator, with shrinkage parameter lambda of min and 1se. The highest internal and external validation result values are highlighted in bold font.

**Table 4:** Prediction model performance and selected predictive markers for eventual aggravation.

Predictive marker selection method		Number of predictors	Selected predictive markers	Model	Internal validation AUC	External validation AUC
LASSO^*^	Min	6	Age + Body temperature + Chest X-ray infiltration + Diabetes + Heart failure + CKD	LR	0.786	0.787
				RF	0.786	0.773
				SVM	0.787	0.791
				KNN	0.718	0.641
				XGBoost	0.757	0.719
				DNN	**0.802**	0.772
	1se	2	Age + Chest X-ray infiltration	LR	0.757	**0.804**
				RF	0.675	0.751
				SVM	0.755	0.773
				KNN	0.711	0.690
				XGBoost	0.693	0.744
				DNN	0.741	0.675
Stepwise regression	9	Age + Body temperature + Chest X-ray infiltration + CKD + Smoking + CND + Myalgia + BMI + Fatigue/Malaise		LR	0.794	0.776
				RF	0.775	0.708
				SVM	0.768	0.762
				KNN	0.733	0.674
				XGBoost	0.793	0.737
				DNN	0.796	0.723

^*^LASSO, Least absolute shrinkage and selection operator, with shrinkage parameter lambda of min and 1se. The highest internal and external validation result values are highlighted in bold font.

In the internal validation for predicting 5 days aggravation, the highest-performing model was the LR model with variables selected via stepwise selection methods. This model, which incorporated 10 predictive markers, namely, age, chest X-ray infiltration, body temperature, smoking status, myalgia, CKD, CND, malignancy, heart failure, and CLD, achieved an AUC of 0.828. For predicting eventual aggravation, the most effective model was a DNN using variables selected from LASSO (min), incorporating 6 predictive markers: age, body temperature, chest X-ray infiltration, diabetes, heart failure, and CKD. This model achieved an AUC of 0.802.

In the external validation for predicting 5 days aggravation, the most effective model was the XGBoost model with variables selected using LASSO (1se). This model, which included 4 predictive markers (age, chest X-ray infiltration, heart rate, and CKD), achieved an AUC of 0.829. For eventual aggravation, the best-performing model was the LR model with variables selected from LASSO (1se), including only 2 predictive markers: age and chest X-ray infiltration. This model demonstrated a promising AUC of 0.804.

This analysis indicated that models predicting 5 days aggravation consistently outperformed those for eventual aggravation in both internal and external validations. Notably, fewer predictive markers were selected for eventual aggravation prediction. Additionally, external validation emphasized the significance of robust key variables such as age and chest X-ray infiltration, underscoring their consistent importance across different hospital settings. To further understand the feature importance of the best-performing models in the external validation datasets, we conducted a Shapley Additive exPlanations (SHAP) analysis for various machine learning methods. For the LR model in particular, we utilized a detailed forest plot analysis, presented in Supplementary Figure S6. This figure highlights that age and chest X-ray infiltration are the key predictors in our prediction models for forecasting both 5 days and eventual patient aggravation. Their high coefficient estimates and impact values underscore their predictive power. Additionally, chronic kidney disease (CKD) and heart failure significantly influence predictions, particularly for 5 days aggravation. Although chronic neurological disorder (CND) appears as a less prominent predictor, it still plays a discernible role in the models, particularly within the Boramae dataset. Other factors such as smoking, body temperature, and myalgia are included in the models but have a minor predictive influence compared to the main clinical markers. These analyses provided deeper insights into the contribution of each variable to the predictive capabilities of the models.

## Discussion

This study investigated the clinical course of 1,696 patients with mild-to-moderate clinical severity on admission who were hospitalized because of laboratory-confirmed COVID-19 infection. Among patients with mild-to-moderate clinical severity on admission, 11.2% were aggravated, and 30% of aggravated patients died of COVID-19. These aggravation rates and CFRs are higher compared to previous reports (18). In a meta-analysis to estimate the CFR of COVID-19, the pooled CFR of COVID-19 in the general population was 1.0%, and among hospitalized patients it was 13.0% (18). In another study analyzing Korean patients diagnosed with COVID-19 between January 21 and May 31, 2020, it was reported that among mild patients—those with no limitation on daily activities or not requiring supplemental oxygen therapy on admission—only 0.4% of patients experienced disease progression, and none had died from the illness as of day 28 (4). Such a higher aggravation rate and CFR in the study population can be explained by differences in age and prevalence of comorbidities. The patient demographics in the study differed significantly from those in a previous investigation. The study cohort had a higher median age, at 51 years compared to 40 years in the prior study. Furthermore, a 2–3 times higher prevalence of significant comorbidities was observed in the study: 13.6% versus 6.3% for diabetes, 23.4% versus 13% for hypertension, and 4.4% versus 1.4% for dementia. It should be noted that the data are derived from the Daegu MediCity database, which primarily consists of patients diagnosed intensively in February 2020, a time when the medical system was overwhelmed, thereby possibly affecting the outcomes. These insights underscore the necessity for vigilant follow-up of individuals presenting mild symptoms upon admission, given that prognosis can fluctuate based on varying risk factors.

Few natural history studies have reported the clinical progression of patients with COVID-19. In this study, 50% of aggravated cases worsened within the first 2 days of admission, and 75% worsened within 5 days. In all, 86% of fatalities occurred among patients who worsened within 5 days, and 94.8% occurred among those who aggravated within 8 days of hospitalization. These findings are consistent with those of previous studies. In an early study of 138 patients hospitalized in Wuhan for pneumonia due to COVID-19, the median time from the first symptom to dyspnea was 5 days, and that of ARDS was 8 days (12). In another study, the median time from the first symptom to dyspnea was 5 days, and the median time to ARDS was 8 days (13). In addition, the progression pattern in this study did not differ according to comorbidities, such as diabetes, hypertension, heart failure, chronic heart disease, malignancy, dementia, and CKD. As most cases worsen within a span of 5 to 8 days, it is important to closely monitor the patient’s condition during this period.

In this study, variables such as age, chest X-ray infiltration, body temperature, BMI, smoking, myalgia, diabetes, heart failure, hypertension, CKD, CND, malignancy, and CLD were identified as being associated with severe outcomes in COVID-19 patients. These findings are consistent with those in the existing literature. For instance, a study utilizing the OpenSAFELY health analytics platform, a UK-based electronic health record system, identified key predictors of COVID-19-related mortality such as older age, male sex, and deprivation and comorbidities including diabetes, cardiovascular disease, respiratory disease (including severe asthma), obesity, a history of hematological malignancy or other recent cancers, kidney, liver, and neurological diseases, and autoimmune conditions (8). Similar findings have been reported in large studies and meta-analyses involving Chinese patients and other international datasets (10, 21).

Although COPD and asthma have been related to COVID-19 exacerbation, our findings did not suggest COPD and asthma as significant risk factors for COVID-19 exacerbation. These results align with several early studies that reported a lower-than-expected prevalence of these conditions in hospitalized COVID-19 patients (22, 23). While COPD is generally linked to poorer outcomes, the lack of association with asthma in our cohort may be due to protective factors such as inhaled corticosteroid usage, a type 2 immune response, reduced ACE2 expression, and eosinophil accumulation. Some studies have reported that regular use of inhaled corticosteroids for asthma management might reduce the severity of COVID-19 by controlling inflammation in the airways (22–25). Asthma is often associated with a type 2 immune response, which may offer some protection against the type 1 immune response that is predominant in COVID-19 (22). The SARS-CoV-2 virus, which causes COVID-19, uses ACE2 receptors to enter human cells. Some studies suggest that patients with asthma, particularly those on certain medications, might have reduced ACE2 expression in their airway cells, potentially reducing their susceptibility to the virus (22, 26, 27). Asthma is characterized by an accumulation of eosinophils (a type of white blood cell) in the airways. The role of eosinophils in COVID-19 is not fully understood, but there is speculation that they play a protective role in mitigating the severity of COVID-19 (22, 28).

Another point of divergence is the non-selection of cardiovascular disease as a predictive marker in this model, despite its frequent citation in the literature as a significant risk factor for poor COVID-19 outcomes. This is likely attributable to the separate categorization of cardiovascular disease and heart failure and the frequent comorbidity of cardiovascular disease with diabetes and hypertension in this study. This comorbidity could potentially diminish the statistical influence of cardiovascular disease as a separate risk factor.

Furthermore, autoimmune and chronic hematologic diseases were not selected as predictive markers in this study. The small sample size for these conditions may be a contributing factor. Previous reports have suggested that the prognosis of patients with autoimmune diseases may depend on their specific treatment. For example, glucocorticoids have been associated with worse COVID-19 outcomes, whereas anti-TNF therapies appear to reduce the risk of hospitalization (10, 29). It should be noted that this dataset lacked information on the treatment regimens for these comorbid conditions, limiting the possibility of exploring this further.

We also developed models for predicting aggravation within 5 days among patients with mild-to-moderate clinical severity, achieving a predictive power of 0.828. Developed models indicated that age, chest X-ray infiltration, body temperature, current smoking status, underlying CKD, CND, malignancy, heart failure, myalgia, and CLD were risk factors associated with aggravation within 5 days after admission. The prediction model for eventual aggravation included nine predictive markers: age, body temperature, BMI, chest X-ray infiltration, smoking, myalgia, fatigue/malaise, underlying CKD, and CND. These variables have been reported as important predictors in previous studies (17, 30–32).

This study has several limitations. First, the exclusion of laboratory data such as C-reactive protein and D-dimer levels from the analysis was inevitable due to non-uniform scale units across different centers and the limited number of patients undergoing these tests. Despite this, we focused on utilizing clinical observations and readily accessible data to predict disease exacerbation, thereby bypassing the need for specialized laboratory data. This approach is particularly valuable in resource-limited settings. While incorporating established predictors such as CRP and D-dimer could enhance a model’s performance, the scope of this study was limited by data availability. The datasets, compiled from the medical records of 10 different institutions in the wake of the Republic of Korea’s first COVID-19 outbreak in Daegu-Kyungpook Province, were missing a significant amount of laboratory data. CRP levels were available for only 277 of the subjects with complete predictors, and no D-dimer levels were available. Therefore, it was not feasible to incorporate these variables into the analysis.

Second, the patients’ symptoms and past medical history were obtained from daily medical practices and were not systematically acquired under the same predetermined protocol. Therefore, there may have been differences in the datasets depending on the center. Nevertheless, the PCA analysis demonstrating no distinct clustering among the datasets from different centers suggests a consistent trend and uniformity in the gathered data, reducing the concerns regarding the potential impacts of this heterogeneity on the findings.

Thirdly, since the data were recorded during the early stages of the COVID-19 pandemic, the predictive model must be validated using contemporary external data to ensure its continued applicability. Moreover, despite the external validations conducted to ascertain the performance of the predictive models, these models should be validated in diverse settings to confirm their robustness and generalizability across different healthcare environments.

## Conclusion

In conclusion, among COVID-19 patients with mild-to-moderate clinical severity on admission, 11.2% aggravated, and 30% of these aggravated patients succumbed. Three-quarters worsened within 5 days after hospitalization, with 86% of fatality cases occurring among those who worsened within 5 days. Age, chest X-ray infiltration, body temperature, current smoking, underlying CKD, CND, malignancy, heart failure, myalgia, and CLD were associated with aggravation within the first 5 days of admission. Close observation during the first 5 days after hospitalization is important to improve the patient’s prognosis. The implications of our findings extend to the broader healthcare management context: clinicians can leverage our prediction model to prioritize patient monitoring, hospital administrators can make strategic decisions in allocating medical resources more effectively, especially in resource-limited settings, and healthcare strategists can develop tailored patient care plans and informed public health policies. These multifaceted applications of our research highlight its value not only in enhancing individual patient care but also in guiding health policy and resource allocation at a broader level, contributing significantly to the overall management of the COVID-19 pandemic.

## Data availability statement

The raw data supporting the conclusions of this article will be made available by the authors, without undue reservation.

## Ethics statement

The studies involving human participants were reviewed and approved by the Institutional Review Board of Kyungpook National University Hospital (KNUH 2020-03-044) and the Institutional Review Board of Seoul National University Boramae Medical Center (IRB No. 30-2020-054). The studies were conducted in accordance with the ethical standards of the local legislation and institutional requirements, as well as the Helsinki Declaration and its later amendments or comparable ethical standards. All participants provided their written informed consent to participate in this study.

## Author contributions

MM and TP: study conceptualization and design. S-WK: data acquisition. HH and CL: data processing and design of the study. MM, HH, and SL: substantive revision. MM and SL: interpretation of the data. MM, HH, CL, SS, and TG: manuscript preparation. HH, SS, and TG: analyses. TP: study supervision. All authors contributed to the article and approved the submitted version.

## Glossary

CFR: Case fatality rate
ARDS: Acute respiratory distress syndrome
BMI: Body mass index
CLD: Chronic liver disease
CKD: Chronic kidney disease
CND: Chronic neurological disease
COPD: Chronic obstructive pulmonary disease
CHD: Chronic hematological disease
LR: Logistic regression
RF: Random forest
SVM: Support vector machines
DNN: Deep neural network
XGBoost: Extreme gradient boosting
KNN: *k*-nearest neighbor
AUC: Area under the curve
ROC: Receiver operating characteristics
