# Supplementary figures and images for: The clinical course of hospitalized COVID-19 patients and aggravation risk prediction models: a retrospective, multi-center Korean cohort study
Source: Front Med (Lausanne). 2024 Jan 4;10:1239789. doi: 10.3389/fmed.2023.1239789 (PMC10794356; doi:10.3389/fmed.2023.1239789)

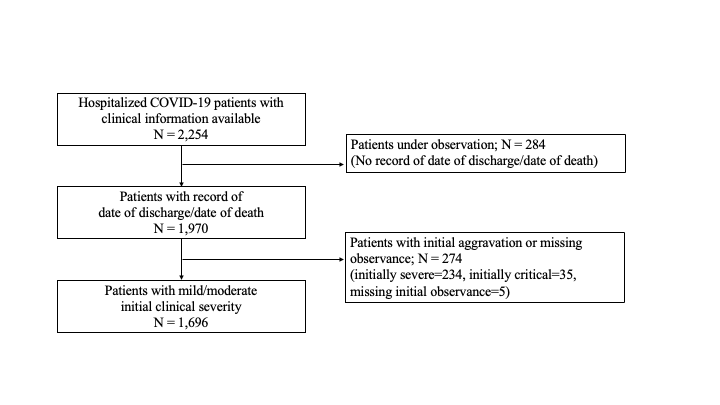

Supplement: Supplementary file 1 [file Image_1.TIFF]
